# Oxygen deficit and H_2_S in hemorrhagic shock in rats

**DOI:** 10.1186/cc11661

**Published:** 2012-10-02

**Authors:** Andry Van de Louw, Philippe Haouzi

**Affiliations:** 1 Pennsylvania State University, College of Medicine, Division of Pulmonary and Critical Care Medicine, Penn State Hershey Medical Center, 500 University Dr., PO Box 850, Hershey, PA 17033, USA

## Abstract

**Introduction:**

Hemorrhagic shock induced O_2 _deficit triggers inflammation and multiple organ failure (MOF). Endogenous H_2_S has been proposed to be involved in MOF since plasma H_2_S concentration appears to increase in various types of shocks and to predict mortality. We tested the hypothesis that H_2_S increases during hemorrhagic shock associated with O_2 _deficit, and that enhancing H_2_S oxidation by hydroxocobalamin could reduce inflammation, O_2 _deficit or mortality.

**Methods:**

We used a urethane anesthetized rat model, where 25 ml/kg of blood was withdrawn over 30 minutes. O_2 _deficit, lactic acid, tumor necrosis factor (TNF)-alpha and H_2_S plasma concentrations (Siegel method) were measured before and after the bleeding protocol in control animals and animals that received 140 mg/kg of hydroxocobalamin. The ability to oxidize exogenous H_2_S of the plasma and supernatants of the kidney and heart homogenates was determined *in vitro*.

**Results:**

We found that withdrawing 25 ml/kg of blood led to an average oxygen deficit of 122 ± 23 ml/kg. This O_2 _deficit was correlated with an increase in the blood lactic acid concentration and mortality. However, the low level of absorbance of the plasma at 670 nm (A_670_), after adding *N, N*-Dimethyl-*p*-phenylenediamine, that is, the method used for H_2_S determination in previous studies, did not reflect the presence of H_2_S, but was a marker of plasma turbidity. There was no difference in plasmatic A_670 _before and after the bleeding protocol, despite the large oxygen deficit. The plasma sampled at the end of bleeding maintained a very large ability to oxidize exogenous H_2_S (high μM), as did the homogenates of hearts and kidneys harvested just after death. Hydroxocobalamin concentrations increased in the blood in the μM range in the vitamin B12 group, and enhanced the ability of plasma and kidneys to oxidize H_2_S. Yet, the survival rate, O_2 _deficit, H_2_S plasma concentration, blood lactic acid and TNF-alpha levels were not different from the control group.

**Conclusions:**

In the presence of a large O_2 _deficit, H_2_S did not increase in the blood in a rat model of untreated hemorrhagic shock. Hydroxocobalamin, while effective against H_2_S *in vitro*, did not affect the hemodynamic profile or outcome in our model.

## Introduction

The severity of a shock secondary to an acute hemorrhage is not simply dictated by the volume of blood loss [1,2]. Rather, the prognosis of a hemorrhagic shock is linked to a cascade of events, occurring during both the phase of bleeding and resuscitation, related to the magnitude of the oxygen deficit [3-6] and the resulting ischemic and post-ischemic inflammatory response [7,8]. Indeed, hemorrhagic shock precipitates inflammatory cascades that comprise the activation of stress transcriptional factors and up-regulation of cytokines synthesis [9,10] leading to multiple organ failure [10]. Among the putative actors involved in the fatal course of an acute hemorrhage induced tissue ischemia/hypoxia, a novel candidate has been recently put forward: endogenous hydrogen sulfide [11,12]. Endogenous H_2_S, a newly described gaso-transmitter [13], has been shown to increase during and following an acute hemorrhage [11] and to act as a powerful pro-inflammatory agent in various animal models [14-17]. In humans, endogenous H_2_S has been proposed 1) to increase in the blood up to 100 μM concentrations during various forms of shock [18] and 2) to be a predictor of survival [18]. Although the mechanism of H_2_S production remains to be clarified in shock, this by-product of cysteine metabolism appears to increase under hypoxic conditions [19,20] and has been more recently suggested to contribute to the response to hypoxia [19,21-23], although this notion has been challenged [24-26]. One of the working hypotheses is that in hypoxic conditions, the level of H_2_S oxidation in the cells and mitochondria is diminished [23]; in turn, the accumulation of this gas was proposed to transduce the physiological response to hypoxia in the vessels or the arterial chemoreceptors [23], but also an unwanted inflammatory response in other tissues [27].

Many questions on the role of H_2_S in hemorrhage, however, remain to be clarified: there are, for instance, many reasons to believe that H_2_S cannot accumulate in the blood [28,29]. Indeed, the view that H_2_S increases in conditions associated with a hemorrhagic shock must be reconciled with the ability of the blood, the cytoplasm of most cells and the mitochondria to oxidize very large amounts of sulfide [29,30], which should prevent H_2_S from rising even at low PO_2 _[24]. One should also reconcile the view that H_2_S concentrations could rise in the body and has deleterious effects with 1) the levels of sulfide found during H_2_S intoxication (see [31] for discussion), which are much lower than those reported in shock [18], and 2) the observations that exogenous H_2_S appears to be beneficial [32-34]. The clinical significance of such a beneficial effect remains the subject of debate [35].

We have recently investigated the effects of cobalt in the form of hydroxocobalamin on H_2_S oxidation [30]. Injection of a large dose of vitamin B12 (at a level similar to that used in cyanide intoxication) dramatically increases the oxidative capacity of the blood and tissues (the kidney and to a lesser extent, the heart) for H_2_S in the rat, possibly via the presence of oxidized cobalt [30]. Acting on H_2_S oxidation in conditions associated with a reduction in oxidative mitochondrial metabolism may, however, represent a way to: 1) test the possible role of endogenous H_2_S in clinically relevant conditions, such as hemorrhage induced tissue ischemia, and 2) evaluate potential novel therapeutic approaches in hemorrhagic shock.

The aim of this study was to determine in a model of untreated hemorrhagic shock in spontaneously breathing urethane anesthetized rats, wherein a large O_2 _deficit can be produced; 1) the putative changes in H_2_S concentration in blood induced by this model of shock and 2) the potential benefit of large doses of vitamin B12 injected before the onset of the hemorrhage. The effects of the presence of μM levels of vitamin B12 in the blood and tissues on the survival at one hour, on the level of lactic acidosis, TNF-alpha and on O_2 _deficit accumulated during and following the period of hemorrhage were investigated, in keeping with the ability of blood and tissues (kidney and heart) to oxidize H_2_S. The possibility of bias accounting for the discrepancy between the low-expected and high-reported changes in H_2_S in humans was also investigated using the same methodological approaches as in published studies [11,16,18]. The hypothesis tested in this study is that H_2_S increases along with inflammatory markers when O_2 _deficit develops during a hemorrhagic shock, and that increasing the oxidative property of the blood and tissues for H_2_S by the presence of vitamin B12 could decrease these markers and improve survival.

## Materials and methods

### Animal preparation

After approval from the Pennsylvania State University College of Medicine Institutional Animal Care and Use Committee, a total of 17 adult Sprague-Dawley rats (470 ± 43 g) were prepared as follows: anesthesia was induced with 3.5% isoflurane in O_2 _followed by intra-peritoneal injection of 1.2 g/kg of urethane (Sigma-Aldrich, St Louis, MO, USA). A polyethylene PE-50 catheter was inserted into the left femoral artery for blood withdrawal and arterial blood pressure (ABP) monitoring (Cybersense, Nicholasville, KY, USA). The animals were tracheostomized and the tracheostomy was connected to a small dead space two-way valve [24]. The inspiratory port of the valve was connected to a calibrated pneumotachograph (Hans Rudolph Inc., KS, USA, 8420 series, Kansas city, MO, USA) to measure inspiratory flow. The rats were breathing spontaneously in room air during the entire protocol. Their body temperature was monitored using a rectal probe and was kept at 35 to 36°C throughout the surgery and the hypovolemia using a pad heated at a constant temperature.

### Protocol

Immediately after surgery, the rats received an intraperitoneal (I.P.) injection of either 140 mg/kg hydroxocobalamin (vitamin B12a, Sigma-Aldrich, 60 mg/ml) in saline (vitamin B12 group, *n *= 9), or an equivalent volume of saline (2.3 ml/kg, control group, *n *= 8). Each rat receiving saline or vitamin B12 was randomly chosen among a homogenous group of rats of similar age and weight. Thirty minutes after I.P. injection, hemorrhage was initiated by withdrawing 2.5 ml/100 g of blood over about 30 minutes as follows: 0.5 ml/100 g were withdrawn over 3 minutes, every 6 minutes (5 sessions). Blood gas analysis and lactate measurements were performed just before and at the end of the hemorrhage period (i-STAT-1 blood gas analyser, Abaxis, Union City, CA, USA). The first and last samples of blood withdrawn were also used for H_2_S and vitamin B12 determinations (see below). Plasma was collected by centrifuging the blood 15 minutes at 13,000 rpm, and then frozen for the determination of TNF-alpha levels and of the ability of the plasma to oxidize H_2_S (see below). No fluid was administered except for flushing the arterial catheter with a fixed volume of 0.2 ml of heparinized saline after each period of bleeding. After the hemorrhage period, data were continuously recorded until the death of the animal.

### Measurements and data analysis

The inspiratory flow ($\dot{\text{V}}$) and arterial pressure signals were digitized by analog-to-digital converter at 200 Hz (LabView 8.5, National Instruments, Austin, TX, USA). Analysis of data was performed offline using Powerchart software (Chart 5, AD Instruments, Colorado Springs, CO, USA). Breathing frequency (f) and tidal volume (VT) were respectively determined using peak detection and integration of the inspiratory flow signal. Minute ventilation ($\dot{\text{V}}\text{I}$) was computed in body temperature and pressure saturated (BTPS) conditions as f × VT.

In 10 animals (4 controls and 6 hydroxocobalamin-treated rats), a 7 ml mixing chamber was connected to the expiratory port of the valve, where mixed expiratory gas composition was continuously sampled and analyzed (GEMINI, CWE Inc., Ardmore, PA, USA). O_2 _uptake ($\dot{\text{V}}{\text{O}}_{\text{2}}$) was computed in standard temperature and pressure, dry (STPD) condition using $\dot{\text{V}}\text{I}$, the inspiratory and expiratory fractions of O_2 _and CO_2_. $\dot{\text{V}}\text{E}$ was computed as $\dot{\text{V}}{\text{I}}_{\text{BTPS}}$ (1-FIO_2_-FICO_2_/1-FEO_2_-FECO_2_) and $\dot{\text{V}}{\text{O}}_{\text{2}}$ as $\left(\dot{\text{V}}{\text{I}}_{\text{STPD}}\;\text{FE}{\text{O}}_{\text{2}}\right)-\left(\dot{\text{V}}{\text{E}}_{\text{STPD}}\text{FE}{\text{O}}_{\text{2}}\right)$. The same approach was used to calculate $\dot{\text{V}}\text{C}{\text{O}}_{2}$ as $\dot{\text{V}}{\text{E}}_{\text{STPD}}\text{FE}{\text{O}}_{\text{2}}$. Oxygen deficit (ml/kg) was computed as the integral of difference between pre-hemorrhage $\dot{\text{V}}{\text{O}}_{\text{2}}$ (averaged over five minutes) and $\dot{\text{V}}{\text{O}}_{\text{2}}\left(\text{t}\right)$ throughout the hemorrhagic period, then until death occurred. All signals were also displayed on line for monitoring.

Hydroxocobalamin concentrations in plasma and tissue homogenates were determined by spectrophotometric reading of the plasma at 525 nm (DU 530, Beckman Coulter, Danvers, MA, USA) as previously described [30].

The methylene blue method [36] was used for H_2_S measurements in the plasma since this method was the one chosen in previous studies to establish the blood levels of H_2_S increases in humans during shock [16,18]. We followed a similar protocol: after centrifuging 2.5 ml of blood at 13,000 rpm for five minutes, 1 ml of plasma was collected and 0.4 ml of zinc acetate (1%) was added to the plasma to trap H_2_S. Then, 100 μl of a 20 mM solution of *N, N*-Dimethyl-*p*-phenylenediamine sulfate (Sigma, St Louis, MO, USA) in 7.2 N hydrochloric acid (Sigma), and 100 μl of a 30 mM iron chloride solution (Sigma) in 1.2 N hydrochloric acid were added to the plasma, producing a blue dye proportional to H_2_S concentration. Throughout the procedure, every precaution was taken to prevent the samples from being in contact with air: the blood was collected in syringes that were immediately capped, and was then transferred to vials, which were completely filled, capped and centrifuged. After centrifugation, the plasma was collected and the reagents were immediately added. The same procedure (including centrifugation) was applied to phosphate-buffered saline (PBS) solution containing a known amount of H_2_S (concentration 100 μM). The H_2_S concentration was obtained after 20 minutes by adding 0.5 ml of trichloroacetic acid (TCA) 10% (to remove the proteins), centrifuging the solutions (10 minutes at 13,000 rpm) and reading the absorbance of the supernatants at 670 nanometers (spectrophotometer Beckman Coulter DU 530). A calibration curve for H_2_S concentration was established, and for each experiment, a PBS solution was used as the blank.

In order to assess the ability of the plasma to oxidize exogenous H_2_S, 0.6 ml of plasma was mixed with 0.6 ml of PBS. NaHS (sodium hydrosulfide hydrate, Sigma-Aldrich) solution was added to the diluted plasma, so that the final concentration of H_2_S was 100 μM. The vials were entirely filled with diluted plasma and as soon as H_2_S was added, the vials were capped to avoid contact with air. Two minutes later, residual H_2_S concentration was measured at ambient barometric pressure after application of 0.5 ml of TCA 10% (Sigma-Aldrich) prior to the final centrifugation (13,000 rpm for 10 minutes), and the absorbance was read at 670 nm.

The same approach was used with the homogenates of hearts and kidneys harvested immediately after cardiac arrest. The organs were thoroughly rinsed in PBS, frozen in liquid nitrogen and stored at -80°C for later analysis. They were then thawed in ambient air, weighed and homogenized (Tissue-Tearor, Biospec, Bartlesville, OK, USA) in PBS (50% w/v for kidney, 25% w/v for heart). The homogenates were then centrifuged at 13,000 rpm for 15 minutes; 1.35 ml of supernatant was collected and mixed with the corresponding volume of NaHS solution to obtain a final concentration of 100 μM following the same procedure as for the plasma. Again, the vials were entirely filled with supernatant and as soon as H_2_S was added, the vials were capped to avoid contact with air. Residual H_2_S concentration was determined after two minutes.

As whole blood is known to readily oxidize H_2_S, we also sought to determine the resolution of the methylene blue method applied to the blood by adding known concentrations of H_2_S in fresh blood from three sham rats and then measuring, for two minutes, H_2_S concentrations using the very same procedure. Again the same measurements were made with the same timing using PBS solution.

TNF-alpha was measured in the plasma samples in duplicates using an ELISA OptEIA kit (BD Biosciences, San Diego, CA, USA).

### Statistical analysis

All results are presented as mean ± SD. All parameters were compared between pre-bleeding and post-bleeding periods using a one-way ANOVA in each group. ABP, $\dot{\text{V}}\text{I}$ and $\dot{\text{V}}{\text{O}}_{\text{2}}$ were also analyzed in each group before and after each of the five bleeding periods using ANOVA for repeated measurements; *post-hoc *comparisons were performed using a Bonferroni correction (SigmaStat 2.0, SPSS Inc, San Jose, CA, USA). Finally, the control and vitamin B12 groups were compared with ANOVA, while survival rates were compared between the two groups using a logrank test [37]. For all comparisons, *P *< 0.05 was considered statistically significant.

## Results

### The shock model

#### Control animals (*n *= 8)

**Figure 1:**
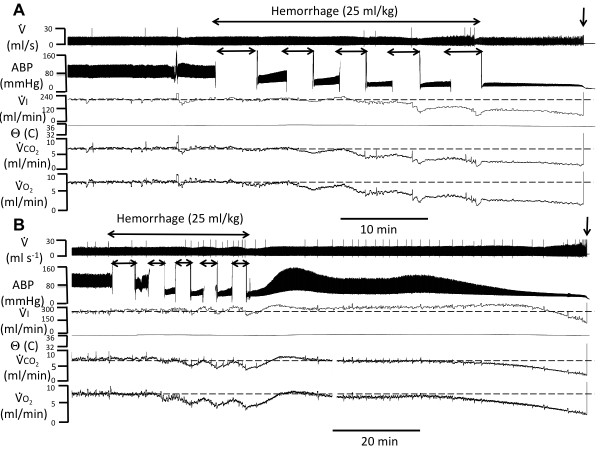
**Examples of recordings in two rats during and following acute hemorrhage**. The breath-by-breath inspiratory flow ($\dot{\text{V}}$), arterial blood pressure (ABP), minute ventilation ($\dot{\text{V}}\text{I}$), body temperature (θ), carbon dioxide production ($\dot{\text{V}}\text{C}{\text{O}}_{2}$) and oxygen uptake ($\dot{\text{V}}{\text{O}}_{\text{2}}$) are displayed. Interruptions in ABP recording are due to blood withdrawal during each of the bleeding periods. Note the drop in arterial pressure, minute ventilation, $\dot{\text{V}}{\text{O}}_{\text{2}}$ and $\dot{\text{V}}\text{C}{\text{O}}_{2}$, during each bleeding period (see text for more details). At the end of the five bleeding periods, all variables continued to either decrease slowly until death occurred (panel **A**, this response was observed in three out of eight control rats), or ABP, $\dot{\text{V}}\text{I}$, $\dot{\text{V}}\text{C}{\text{O}}_{2}$ and $\dot{\text{V}}{\text{O}}_{\text{2}}$ rose transiently before subsiding again, leading to a fatal outcome (panel **B**, this response was observed in five out of eight control rats). The vertical arrow corresponds to the final cardio-respiratory arrest.

**Figure 2:**
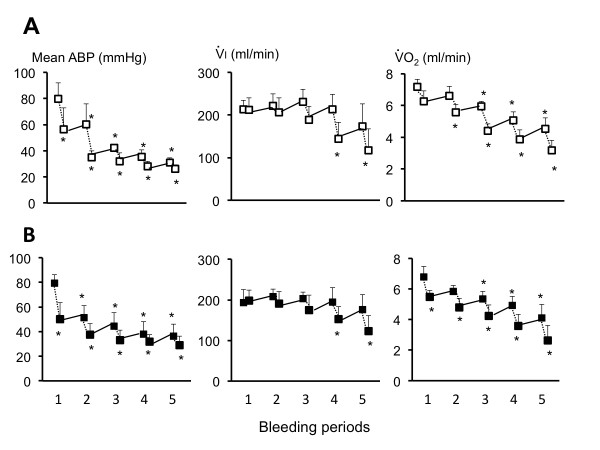
**ABP, $\dot{\text{V}}\text{I}$ and $\dot{\text{V}}{\text{O}}_{\text{2}}$ before and after each bleeding periods, in control (A) and vitamin B12 treated rats (B)**. The dashed lines represent the bleeding periods. ABP, $\dot{\text{V}}\text{I}$ and $\dot{\text{V}}{\text{O}}_{\text{2}}$ dropped during each blood withdrawal, rising again when bleeding was stopped but without reaching their previous values. There was no difference between control and vitamin B12-treated rats for either parameter. *P < 0.05 *vs *pre-bleeding baseline (period 1).

**Figure 3:**
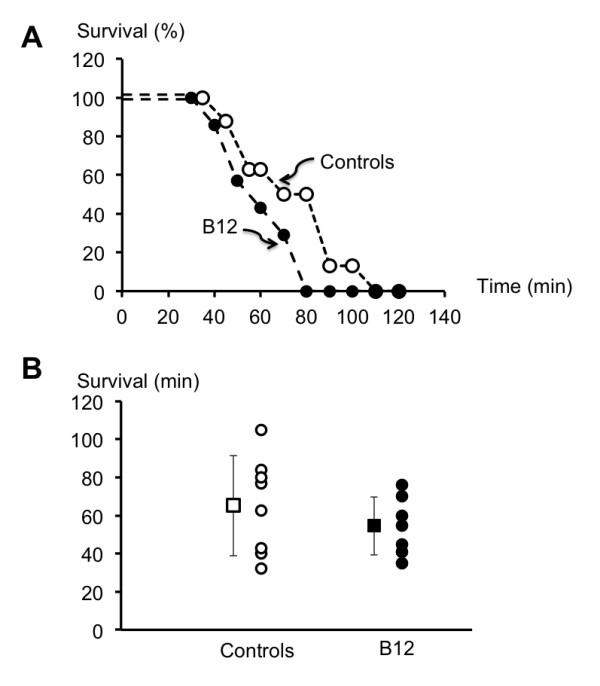
**Survival rates**. Panel **A**, survival rate (in %) in the control (open circles) and vitamin B12-treated rats (closed circles). Time zero corresponds to the onset of the bleeding protocol. All rats survived the bleeding period (30 minutes), about 50% were still alive after 60 minutes, and no rats survived after 120 minutes. There was no difference in the survival rates between the two groups (logrank test). Panel **B **displays the individual survival times for the two groups. There was no significant difference in the mean survival time between control and vitamin B12 treated rats (65 ± 26 *vs *59 ± 26 min).

**Figure 4:**
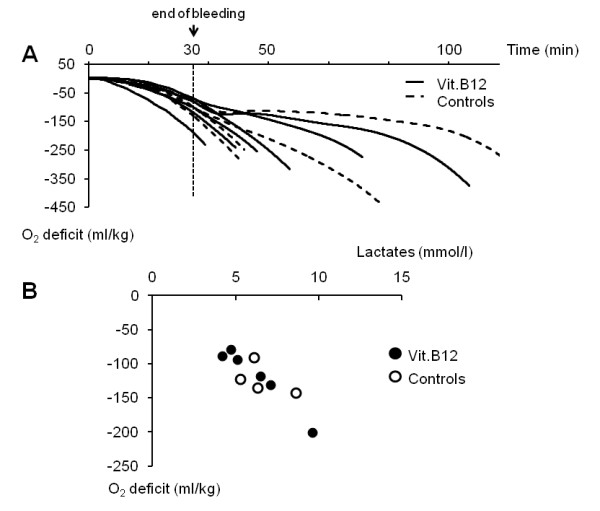
**Oxygen deficit in control vs treated rats**. Panel **A**, time course of O_2 _deficit in four control (dashed lines) and six vitamin B12-treated (continuous lines) rats, from the onset of bleeding (time 0) to death. In all rats, O_2 _deficit accumulated continuously during the bleeding period reaching 120 ml/kg at the end of the bleeding protocol (vertical arrow). Following the bleeding period, O_2 _deficit continued to accumulate in a ramp-like fashion, while in the rats that survived much longer, O_2 _deficit remained constant. There was no significant difference in the time course of O_2 _deficit between control and vitamin B12-treated rats. Panel **B**, relationship between O_2 _deficit and plasma lactate levels at the end of bleeding periods in control (open circles) and vitamin B12-treated (closed circles) rats (r^2 ^= 0.79).

Figure 1 displays recordings of the response to the hemorrhage protocol in two different rats, while Table 1 reports the averaged data. Hemodynamic, ventilatory and metabolic responses were qualitatively similar in all control rats. Typically, each of the five three-minute bleeding periods induced a drop in arterial pressure, minute ventilation, $\dot{\text{V}}{\text{O}}_{\text{2}}$ and $\dot{\text{V}}\text{C}{\text{O}}_{2}$. Between the bleeding periods, all the parameters tended to return progressively to their baseline values (Figures 1 and 2). This recovery was interrupted by the subsequent bleeding periods repeated after six minutes and was blunted over time. At the end of the bleeding periods (30 minutes), mean ABP, $\dot{\text{V}}\text{I}$, $\dot{\text{V}}{\text{O}}_{\text{2}}$ and $\dot{\text{V}}\text{C}{\text{O}}_{2}$ were significantly reduced by 68%, 44%, 56% and 51% respectively (see actual data in Table 1). Blood lactic acid increased significantly (*P *= 0.001) (Table 1). Two different profile patterns (Figure 1) were observed following the bleeding procedure: in five animals, arterial pressure increased slowly towards pre-bleeding levels before subsiding again until death occurred from primary respiratory or cardiac arrest, within two hours following the onset of bleeding. In the three remaining animals, arterial pressure, minute ventilation, $\dot{\text{V}}{\text{O}}_{\text{2}}$ and $\dot{\text{V}}\text{C}{\text{O}}_{2}$ continued to decrease until death (Figure 1), which occurred within one hour. The survival rate *vs*. time is shown in Figure 3. The O_2 _deficit which averaged 122 ± 23 ml/kg at the end of the bleeding period (Figure 4 and Table 1) reached 338 ± 88 ml/kg at the moment of death.

**Table 1:** Hemodynamic and metabolic variables before and at the end of the bleeding period.

	Control (*n *= 8)		Vitamin B12 (*n *= 9)	
	**Pre-bleeding**	**End of bleeding**	**Pre-bleeding**	**End of bleeding**
**Mean ABP (mmHg)**	80 ± 12	26 ± 3*	79 ± 7	29 ± 7*
**Minute ventilation (ml/min)**	212 ± 22	118 ± 50*	194 ± 31	123 ± 39*
$\dot{V}O_{2}\left(\mathrm{ml}/\min\right)$	7.18 ± 0.45	3.19 ± 0.60*	6.79 ± 0.67	2.63 ± 0.97*
$\dot{V}CO_{2}\left(\mathrm{ml}/\min\right)$	6.55 ± 0.60	3.23 ± 0.70*	6.42 ± 1.14	2.66 ± 1.03*
**Lactates (mM/l)**	1.88 ± 0.50	6.35 ± 1.44*	1.53 ± 0.21**	6.63 ± 2.09*
**PO_2 _(mmHg)**	81 ± 5	90 ± 11	72 ± 9**	86 ± 11*
**PCO_2 _(mmHg)**	36 ± 4	31 ± 7	33 ± 8	29 ± 5
**TNF-alpha (pg/ml)**	-	1,301 ± 1,175	-	732 ± 869
**O_2 _deficit (ml/kg)**	-	122 ± 23	-	118 ± 45

Values are mean ± SD. *P < 0.05 *vs *pre-bleeding values. ** *P *< 0.05 *vs *control rats.

#### Vitamin B12 treated rats (*n *= 9)

**Figure 5:**
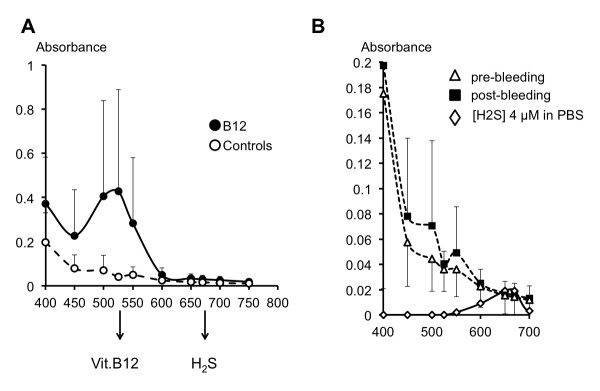
**Absorbance of the plasma and H_2_S concentrations**. Panel **A**, absorbance spectra of the plasma collected at the end of the overall bleeding period in control (open circles) and vitamin B12-treated (closed circles) rats. There was a clear peak of absorbance at 525 nm in vitamin B12-treated rats corresponding to a vitamin B12 plasma concentration of 185 ± 216 μM/l. No peak was observed in any of the control rats. At 670 nm, that is, the absorbance wavelength of the methylene blue, no peak was observed, neither in control nor in the vitamin B12-treated rats. Panel **B**, absorbance spectra, between 400 and 700 nm, of the plasma of the control rats, before (open triangles) and at the end of the bleeding period (closed squares). The observed absorbance values at 670 nm would theoretically correspond to a H_2_S concentration of around 4 μM/l in the dilute plasma (or 8 μM/l in the plasma, see text for additional comments), as illustrated using a control solution (phosphate-buffered saline, PBS) containing H_2_S (4 μM) (open diamonds). The lack of peak of absorbance in the plasma at 670 nm along with the pattern of absorbance over the visible spectrum (continuous decrease of absorbance from 400 nm) strongly suggest that it is the turbidity of the medium which could account for this apparent presence of H_2_S in the plasma.

The absorbance spectra of the plasma of the animals treated with vitamin B12 clearly showed a peak of absorbance at 525 nm (Figure 5A), corresponding to a concentration of 185 ± 216 μM/l. No peak of absorbance at 525 nm was observed in the plasma of any of the control rats (Figure 5A).

As shown in Figure 2 and Table 1, the changes in ABP, minute ventilation, $\dot{\text{V}}{\text{O}}_{\text{2}}$, $\dot{\text{V}}\text{C}{\text{O}}_{2}$ and lactate before, during and after the bleeding periods were similar in the group treated with vitamin B12 and in control animals. The time course of O_2 _deficit was also the same in the two groups of rats (Figure 4A). O_2 _deficit accumulated progressively during the bleeding period, reaching 118 ± 45 at 30 minutes (Table 1, NS *vs *controls, *P *= 0.98). When vitamin B12 and control rats were combined, O_2 _deficit and lactate level at the end of bleeding were significantly correlated (Figure 4B, r^2 ^= 0.79). The O_2 _deficit at the time of death was 265 ± 30 ml/kg (Table 1, NS *vs *controls, *P *= 0.10).

### H_2_S measurements

#### Control animals

According to our standard curve, a concentration of 100 μM H_2_S resulted in an absorbance of 1.41 at 670 nm; the relationship between the concentration of H_2_S and the absorbance was linear up to 3 μM while it was possible to identify the presence of H_2_S at a minimal value of 1.5 μM (absorbance 0.005). We did not find any changes in the level of H_2_S added to PBS which were analyzed following the very same procedure as the blood (including centrifugation): the absorbance of a solution of H_2_S in PBS analyzed immediately after sampling from the "mother" solution dropped by 3.2% following the procedure applied to the blood (*n *= 12). Centrifugation for 10 minutes decreased the absorbance by 1.2%. Absorbance readings of the plasma before the shock averaged 0.014 ± 0.015 (Figure 5B). According to the standard curve, such an absorbance would correspond to a theoretical H_2_S concentration of 8.5 ± 2.9 μM. However, the profile of absorbance over the visible spectrum was markedly different from that of a PBS solution containing H_2_S at a concentration that would reach a similar absorbance at 670 nm; as shown in Figure 5B, the absorbance of the plasma was high at 400 nm and decreased continuously as the wavelength was increased with a lack of peak of absorbance at 670 nm, in major contrast to the PBS solution. In other words, in the absence of absorbance peak at 670 nm, the value of absorbance of 0.014 ± 0.015 in the plasma did not reflect the presence of methylene blue - and thus H_2_S at approximately 8 μM - but should be viewed as a marker of turbidity.

Similar results were found at the end of the bleeding period (when O_2 _deficit reached 122 ± 23 ml/kg) with an absorbance of 0.016 ± 0.009 at 670 nm. No peak was observed and, just like before the bleeding period, a progressive decrease in absorbance from 400 to 700 nm was found (Figure 5B). This profile of absorbance and the lack of peak at 670 nm were observed in every animal with no exception.

#### Vitamin B12 treated animals

There was no significant difference between the absorbance at 670 nm in control animals and following vitamin B12, both prior (0.017 ± 0.009) and following (0.029 ± 0.018) the period of bleeding (Figure 5A). Just like in the control group and in major contrast to the PBS solution, no peak could be identified at 670 nm suggesting that H_2_S concentration in the plasma, if any, could not be higher than a few μM.

#### Oxidation of 100 μM H_2_S by the plasma before and during shock

**Figure 6:**
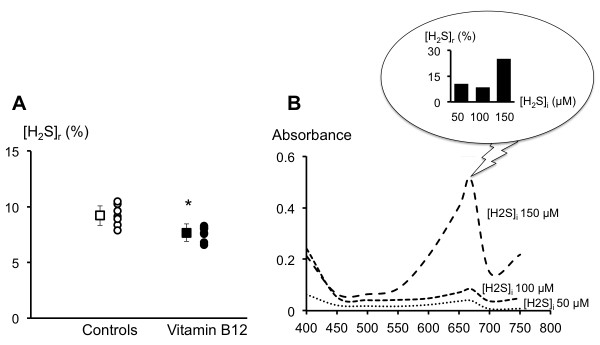
**Plasma and H_2_S**. Panel **A**, residual H_2_S concentration ([H_2_S]_r_), two minutes after addition of 100 μM H_2_S in the plasma of control (open circles) and vitamin B12-treated (closed circles) rats. [H_2_S]_r _is expressed in percentage of the concentration of 100 μM present in a control PBS solution analyzed at the same time as the plasma. Within two minutes, H_2_S concentrations decreased by more than 90% in control and vitamin B12-treated rats respectively, with a significant difference between the two groups (*P < 0.05). Panel **B**, absorbance spectra of H_2_S added to sham rat whole blood at 50, 100 and 150 μM and measured after two minutes. In the inset, the percentage of residual [H_2_S] ([H_2_S]_r_), corresponding to the absorbance at 670 nm, is shown for each initial concentration. Depending of the initial [H_2_S] ([H_2_S]_i_), exogenous H_2_S concentrations decreased between 92 and 75%.

As shown on Figure 6A, two minutes after adding 0.1 ml H_2_S to pre-bleeding plasma to reach a final concentration of 100 μM, residual H_2_S concentration was 9.2 ± 0.9 μM in the control plasma and 7.7 ± 0.8 μM in the plasma of the animals which received vitamin B12 (*P *< 0.01). No change in H_2_S concentration was found in the PBS solution over two minutes. The ability of the plasma to oxidize H_2_S remained unchanged at the end of the bleeding period, with residual plasma H_2_S concentrations of 10.0 ± 1.0 and 7.2 ± 1.7 μM for the control and vitamin B12 groups respectively (*P *< 0.01). For the whole blood, the absorbance spectra of H_2_S added in sham rat blood at three different concentrations (50, 100 and 150 μΜ) is displayed on Figure 6B along with the corresponding residual H_2_S concentrations. Within five minutes, initial H_2_S concentrations in blood of 50,100 and 150 μΜ dropped by 90, 92 and 75% respectively.

#### Oxidation of 100 μM H_2_S by tissue homogenates

**Figure 7:**
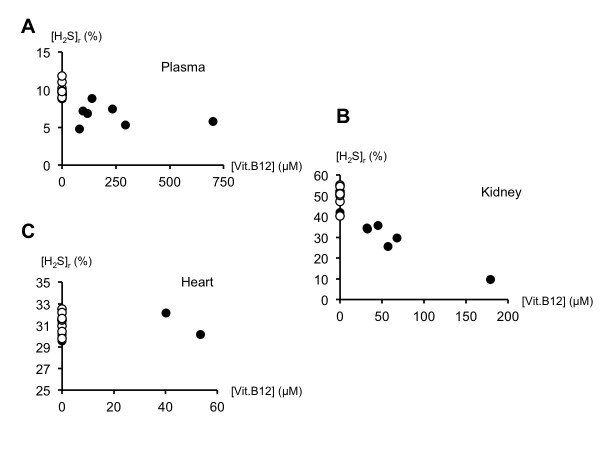
**Ability of plasma and tissue homogenates to oxidize/complex H_2_S**. Individual relationship between vitamin B12 concentration and residual H_2_S concentration ([H_2_S]_r_) determined two minutes after addition of 100 μM H_2_S in the plasma (Panel **A**), in the supernatant of kidney homogenates (Panel **B**) and heart homogenates (Panel **C**) in all the control (open circles) or vitamin B12-treated (closed circles) rats. [H_2_S]_r _is expressed in percentage of the initial concentration of 100 μM present in a control PBS solution analyzed at the same time as the medium. In the presence of vitamin B12, very large amounts of H_2_S can be oxidized both in the plasma and in the kidney. In the heart, the concentration of vitamin B12 was too low to be measured by our method (except for two rats) and no visible effect on high μM concentration of exogenous H_2_S could be identified (see text for additional comments).

As shown on Figure 7B, two minutes after adding 0.15 ml H_2_S to the supernatant of kidney homogenates to reach a final concentration of 100 μM, residual H_2_S concentration was 50.2 ± 4.7 μM in control kidney homogenates and 34.8 ± 12.9 μM in kidney homogenates of the animals which received vitamin B12 (*P *< 0.01). In the same conditions, residual H_2_S concentrations in heart homogenates were 31.3 ± 0.9 and 30.5 ± 0.9 μM for control and vitamin B12-treated rats respectively (Figure 7C, NS). In vitamin B12-treated rats, hydroxocobalamin concentrations were 47 ± 57 μM in kidney homogenates (Figure 7B), and below the threshold of detection (30 μM) in heart homogenates except for two animals (Figure 7C). Concentrations did not change in the PBS solutions.

### Mortality and vitamin B12

There was no difference in time to death (59 ± 26 *vs *65 ± 26 min) or mortality rates over time between vitamin B12 and control rats (Figure 3).

### Plasma TNF-alpha

There was very large intra-group variability but no statistically significant difference in TNF-alpha plasma levels between vitamin B12 treated and control rats (*P *= 0.398, Table 1).

## Discussion

In major contrast to previous reports in humans [18] and in animal models [11], we did not observe any increase in blood H_2_S concentration in a model of lethal untreated hemorrhagic shock in urethane anesthetized rats, despite major O_2 _deficit, hyperlactacidemia and systemic inflammation. In addition, following the bleeding period, the plasma of every animal, as well as the supernatants from the heart and kidney kept a very high ability of oxidizing/complex large (toxic) amounts of sulfide. Finally, following injection of a very large dose of vitamin B12, the ability to oxidize H_2_S by the plasma and the kidney was enhanced in proportion to the local vitamin B12 concentration. However, the survival rate, O_2 _deficit or the various markers of the severity of the shock were not affected by the presence of μM levels of hydroxocobalamin. These findings do not support the hypothesis that endogenous H_2_S does accumulate in the blood or in most tissues and contributes to the severity of hemorrhagic shock induced oxygen deficit [11,18].

### Rat model

To investigate the putative role of endogenous hydrogen sulfide during hemorrhagic shock induced cellular hypoxia, we used the urethane-anesthetized rat as an experimental model. Withdrawing 25 ml/kg of blood within 30 minutes produced a dramatic reduction in ABP, $\dot{\text{V}}{\text{O}}_{\text{2}}$ and $\dot{\text{V}}\text{C}{\text{O}}_{2}$ along with an increase in lactic acid and in the respiratory quotient ratio. This hemorrhage protocol led to a fatal outcome in 50% of the animals within one hour following the bleeding onset, which paralleled the magnitude of O_2 _deficit and hyperlactacidemia. All animals died within two hours. This relatively low survival rate of hemorrhagic shock in rats compared to larger [38,39] or non-anesthetized animals [40] is not unexpected. Indeed, not only anesthesia alone is likely to affect the normal ability of the circulatory and respiratory systems to respond to an acute reduction in volemia [31,41], but urethane, by itself, significantly blunts the normal cardio-vascular regulation [42]. Nevertheless, the fatal outcome we observed in our study appears to be both quantitatively and qualitatively similar to that observed in larger mammals [1,3,5]. A number of characteristics of our model should, however, be discussed. First, O_2 _deficit per kilogram was much larger for a given volume of blood withdrawn than in larger animals, such as pigs [1] or dogs [4,5]. This larger reduction in $\dot{\text{V}}{\text{O}}_{\text{2}}$ during and following the hemorrhage was associated to a higher baseline specific (per kilogram) metabolic rate, typical of small mammals, akin to hypoxia induced metabolic depression [43-46]. More specifically, resting $\dot{\text{V}}{\text{O}}_{\text{2}}$ in our rat model averaged 15 ml/kg (about four times the expected $\dot{\text{V}}{\text{O}}_{\text{2}}$ level in humans), as previously reported [45,47], with a O_2 _deficit reaching 300 ml/kg over a one-hour period, up to three times the deficit reported in sheep or dogs during bleeding protocols leading to similar survival rates [2]. This large $\dot{\text{V}}{\text{O}}_{\text{2}}$ deficit can be accounted for by the magnitude of blood flow redistribution in small *vs *large animals [42,48], a reduction in uncoupling protein activity, specific to small-sized mammals [49] and, eventually, a genuine reduction in oxidative mitochondrial activity resulting in lactic acidemia. Although extremely variable between animals, an elevation in blood level of TNF-alpha was found in all rats at the end of the bleeding period.

It is interesting to note that just like in hypoxia-induced hypometabolism [43] or when unloading venous return [50], ventilation decreased with $\dot{\text{V}}{\text{O}}_{\text{2}}$ and $\dot{\text{V}}\text{C}{\text{O}}_{2}$ but with a relative hyperventilation (Figure 2); this discrepancy between the drop in gas exchange rate and $\dot{\text{V}}\text{I}$ resulted in all animals in a progressive reduction in PaCO_2 _and increase in PaO_2 _(Table 1). Finally, the relative higher values of $\dot{\text{V}}\text{C}{\text{O}}_{2}$ than $\dot{\text{V}}{\text{O}}_{\text{2}}$ at the end of the bleeding protocol are likely to be accounted for by the equimolar transformation of the bicarbonate into CO_2 _(buffering of the developing metabolic acidosis). This could have resulted in a relative increase in CO_2 _output, akin to the rise in the respiratory quotient ratio typical of heavy exercise with hyperlactacidemia [51].

### Hemorrhagic shock and H_2_S

H_2_S has been shown to be present in the plasma at concentrations between 25 and 50 μM and to increase up to 100 μM in humans during various types of shock [18]. Plasma H_2_S concentration in these patients [18] correlates with the severity of the shock. The very presence of H_2_S in the plasma has already been challenged on methodological and physiological grounds [20,28]. Since the proteins present in the blood (hemoglobin) complex and/or catalyze very large amounts of sulfide [20], trivial levels of H_2_S, if any, are expected to be found in the plasma in baseline conditions, as shown by Furne *et al*. [28] and Whitfield *et al*. [20]. Whitfield *et al*. reported no measurable level of H_2_S [20] after addition of 10 μM of H_2_S in rat blood before applying a method similar to that used in the present study. Besides, the levels reported both in baseline conditions and in shock [11,16,18] appear to be higher than those expected to be found during severe H_2_S intoxication [52], wherein mitochondrial activity is inhibited.

The method used to determine H_2_S in previous studies [11,16,18] was developed by Siegel *et al*. [36] and relies on the transformation of one molecule of H_2_S and two molecules of *N, N*-dimethyl-*p*-phenylenediamine into one molecule of methylene blue (MB). H_2_S concentration can then be determined by measuring the absorbance of the solution at 670 nm (methylene blue). One of the limits of this method is directly related to the fact that the absorbance is proportional to the concentration of a given molecule - which color is the complementary of the light wave absorbed - if and only if none of the incident light is scattered by dispersed particles or molecules. The presence of a minimal level of turbidity can alter the absorbance of light at any wavelength irrespective of the actual "color" of the plasma, unless a genuine peak of absorbance can be found between 600 and 700 nm (Figure 5). We found that, even after application of TCA to remove the proteins and multiple centrifugations, a significant absorbance can be found at 670 nm. Using a broader spectrum of wavelengths, one can show that the Beer-Lambert law cannot be applied to identify small concentrations of H_2_S in plasma. This is also illustrated in Figure 5 where the spectrum of absorbance of the plasma after reaction with the reagents to form MB is very different from that of a solution containing H_2_S at the hypothetical concentration corresponding to a similar absorbance. These data suggest that if the method developed by Siegel is to be used in the plasma, determination of H_2_S concentrations based on the absorbance of light at only 670 nm can be misleading, yielding to erroneous findings of H_2_S. This issue has already been highlighted by Hughes *et al*., who reported that the linear dependence of absorbance on the MB concentration was only valid for concentrations of H_2_S much lower than those reported in all these studies; these authors also recommended the use of the spectra of absorbance between 550 and 700 nm [53]. Using a different method, based on the monobromobimane derivatization [54], Tokuda *et al*. reported H_2_S plasma concentrations ranging at best between 2 to 4 μM, consistent with the present results, and more importantly that H_2_S levels decreased, if anything, in endotoxic shock in mice [55]. Volatilization of H_2_S observed by De Leon *et al*. [56] when samples are left in open air is unlikely to have occurred (see Method and Result sections); since we took the precaution to entirely fill all the vials and to cap them immediately, to prevent any significant volatilization. The procedure used for plasma H_2_S measurements (including centrifugation) was applied as well to PBS solutions containing a known amount on H_2_S (concentration 100 μM), and the level of H_2_S was not affected. In addition, we previously found that using this procedure, H_2_S concentration (in PBS or saline solution) remained stable with a few percent drop in concentration over one hour [30]. DeLeon *et al*. reported similar results when they took the precaution to close their chambers [56]. For the determination of the ability of the plasma and tissues to oxidize exogenous H_2_S, PBS solutions containing the same initial amount of H_2_S were analyzed at the very same time as the plasma or supernatant; H_2_S concentrations were unchanged in PBS within the two-minute period we chose for our determination and all results have been expressed in percentage of the concentration of 100 μM present in the PBS solution analyzed at the same time and following the same procedure.

### Endogenous H_2_S in the tissues, vitamin B12 and hemorrhagic shock

There is a spontaneous oxidation/complexation of H_2_S in the plasma and the supernatant of tissues, which was enhanced by μM concentrations of vitamin B12. The latter was obtained following intraperitoneal injection of vitamin B12 at a dose used during cyanide intoxication (10^6 ^times the normal daily intake), as previously reported [46,57,58]. The kidney and heart were chosen as they are among the most important organs exposed to the consequences of hemorrhage-induced ischemia. In addition, our previous study [30] showed that the ability of tissue homogenates of these organs to oxidize H_2_S was clearly enhanced in vitamin B12-treated rats. In that previous study, we found that vitamin B12 could oxidize large amounts of H_2_S in direct relation to its concentration, likely due to the presence of oxidized cobalt [30]. We could establish that the presence of 10 μM vitamin B12 was able to oxidize 20% of a 100 μM solution of H_2_S; at 50 μM, about 80% of the H_2_S was oxidized within five minutes. This is consistent with our present results, where vitamin B12 concentrations in the plasma were found to be about 180 μM and could decrease the exogenous H_2_S levels to 7.7 ± 0.8 μM (significantly lower than control plasma; initial concentration 100 μM), after two minutes. Similarly, about 50 μM of hydroxocobalamin were found in our kidney homogenates, which in turn decreased H_2_S concentrations by 65% (*vs *only 50% in control; initial concentration 100 μM). For the heart and, very likely, for some other tissues, the spectrophotometric method of detection of vitamin B12 was not sufficient to demonstrate the presence of vitamin B12 (the threshold is about 30 μM [30]). Incidentally, pM - and not μM - concentrations of hydroxocobalamin are expected to be present in the body [59,60]; therefore, with the methodology used in the present study we were unable to demonstrate whether low μM concentrations of vitamin B12 were able to oxidize H_2_S at the concentrations likely to be present in the heart [28]. This is a very important point to consider as our present results did not show that vitamin B12 was absent from the heart or could not oxidize sulfide, but within the very poor resolution of our method, no reliable conclusion could be drawn.

Hypoxic conditions have been proposed to decrease H_2_S oxidation resulting in the accumulation of H_2_S [23]. This increase in H_2_S concentrations may, however, occur only if PO_2 _decreases to extremely low levels [24], similar to those expected to be found in the vicinity of the mitochondria, suggesting to Olson [23,61] that the site of action for endogenous H_2_S can only be the mitochondria. Studies trying to establish the actual amount of H_2_S present and endogenously produced revealed that at best nM changes in H_2_S concentrations can be observed [46]. We speculate that low levels of vitamin B12 could still be able to decrease such concentration of H_2_S in our study [30].

The present study did not address the effect of reperfusion, wherein production of cytokines and oxidative stress are prominent. Patients or animal models who showed an increase in plasma H_2_S concentrations [16,18] were resuscitated, and the question of the putative role of endogenous H_2_S will need to be tested during the critical period of reperfusion.

## Conclusions

There is no evidence that H_2_S can accumulate in the high micromolar range in the blood and tissues (extravascular compartment) during a lethal form of hemorrhagic shock. The presence of cobalt (hydroxocobalamin) did not affect any of the outcomes of the shock. These results imply that H_2_S in the blood cannot be used as a marker of hemorrhagic shock. The hypothesis that H_2_S could accumulate during hemorrhagic induced tissular hypoxia must be reconciled with the ability of tissues to oxidize H_2_S.

## Key messages

• Even during a severe form of hemorrhagic shock in the rat, where a major O_2 _deficit is present, there is no evidence for an increase in H_2_S concentration in the blood.

• Injection of high doses of hydroxocobalamin, although enhancing the ability of blood and kidneys to oxidize exogenous H_2_S *in vitro*, does not improve the survival, O_2 _deficit, lactacidemia or TNF-alpha levels in this model of shock.

## Abbreviations

ABP: arterial blood pressure; BTPS: body temperature and pressure saturated; f: breathing frequency; I.P.: intraperitoneal; MB: methylene blue; MOF: multiple organ failure; NS: non-significant; PBS: phosphate buffer saline; STPD: standard temperature and pressure: dry; TCA: trichloroacetic acid; $\dot{\text{V}}$: inspiratory flow; $\dot{\text{V}}\text{I}$: minute ventilation; $\dot{\text{V}}\text{C}{\text{O}}_{2}$: carbon dioxide production; $\dot{\text{V}}{\text{O}}_{\text{2}}$: oxygen uptake; VT: tidal volume

## Competing interests

The authors declare that they have no competing interests.

## Authors' contributions

AV and PH conceived of the study, performed the animal experiments, analyzed the data and drafted the manuscript. All authors read and approved the final manuscript.
